# The Progressive Mutagenic Effects of Acidic Bile Refluxate in Hypopharyngeal Squamous Cell Carcinogenesis: New Insights

**DOI:** 10.3390/cancers12051064

**Published:** 2020-04-25

**Authors:** Clarence T. Sasaki, Sotirios G. Doukas, Jose Costa, Dimitra P. Vageli

**Affiliations:** 1 The Yale Larynx Laboratory, Department of Surgery, Yale School of Medicine, 310 Cedar Street (BML212), New Haven, CT 06510, USA; 2 Department of Pathology, Yale School of Medicine, New Haven, CT 06510, USA

**Keywords:** bile reflux, NF-*κ*B, DNA damage, hypopharyngeal cancer, head and neck cancer

## Abstract

Cancers of the laryngopharynx represent the most devastating of the head and neck malignancies and additional risk factors are now epidemiologically linked to this disease. Using an in vivo model (*Mus musculus* C57Bl/6J), we provide novel evidence that acidic bile (pH 3.0) progressively promotes invasive cancer in the hypopharynx. Malignant lesions are characterized by increasing: (i) oxidative DNA-damage, (ii) γH2AX expression, (iii) NF-κB activation, and (iv) p53 expression. Histopathological changes observed in murine hypopharyngeal mucosa exposed to acidic bile were preceded by the overexpression of *Tnf*, *Il6*, *Bcl2*, *Egfr*, *Rela*, *Stat3*, and the deregulation of *miR-21*, *miR-155*, *miR-192*, *miR-34a*, *miR-375*, and *miR-451a*. This is the first study to document that acidic bile is carcinogenic in the upper aerodigestive tract. We showed that oxidative DNA-damage produced by acidic bile in combination with NF-κB-related anti-apoptotic deregulation further supports the underlying two-hit hypothesized mechanism. Just as importantly, we reproduced the role of several biomarkers of progression that served as valuable indicators of early neoplasia in our experimental model. These findings provide a sound basis for proposing translational studies in humans by exposing new opportunities for early detection and prevention.

## 1. Introduction

It is estimated that head and neck cancer will account for approximately 53,000 new cases and 10,860 deaths in the United States in 2019 [1]. Laryngopharyngeal tumors that mainly represent squamous cell carcinoma account for almost 50% of all head and neck cancers (HNSCC). From a clinical perspective, these tumors are very aggressive, and therefore causing a high mortality rate, with an overall 5-year survival rate of around 60%. Because of the elevated risk of recurrent disease and lower quality of life, patients with hypopharyngeal cancer carry a 13-fold increased risk of suicide [2], additionally identifying it among the most devastating cancers. Although known risk factors, such as smoking, alcohol consumption, and HPV16 infection have been associated with laryngopharyngeal carcinogenesis [3], additional risk factors, such as laryngopharyngeal reflux (LPR), have also been epidemiologically linked to the disease [4,5]. There is growing evidence that approximately 50% to 86% of patients with gastro-esophageal reflux disease (GERD) present with mixed gastric and bile (duodenal) fluids in their refluxates [6,7,8], implying that bile-containing enterogastric reflux is much more common than previously appreciated [9]. Clinically, it has been shown that bile refluxate frequently reaches the epithelium of the upper aerodigestive tract, supporting its independent role as a causative risk factor in the supraesophageal development and progression of neoplastic events in the laryngopharynx [10].

We recently focused our research on the role of LPR and particularly the role of bile refluxate in laryngopharyngeal carcinogenesis. The carcinogenic potential of bile acids has been previously discussed regarding the colon and esophagus [11,12], identifying NF-κB as a key molecule in bile-related adenocarcinoma of the esophagus [13]. Although, there is evidence to support the clinical association of bile reflux with Barrett’s esophagus [14,15], a known precursor of esophageal adenocarcinoma, the mechanistic role of bile as a carcinogen in esophageal and laryngopharyngeal squamous cell carcinoma is not well established, nor to date is there exploratory evidence to confirm the mutagenic properties of bile refluxate in the development of squamous cell carcinoma in this setting [4,5,12]. Using a preclinical model, we have described that a 45-day exposure of laryngopharyngeal mucosa to acidic bile is capable of inducing premalignant squamous epithelial lesions in murine laryngopharyngeal mucosa [16,17]. Importantly, the epithelial lesions are preceded by enhanced activation of cytoplasmic and nuclear NF-κB promoting significant molecular alterations of cancer-related genes, such as *Bcl2*, *Egfr*, *Stat3*, *Tnf*, and *Il6*, and miRNA markers, such as *miR-21*, *miR-155*, *miR-192*, *miR-375*, *miR-451a*, and *miR-34a*. We also recently described and documented that these acidic bile-induced early pre-neoplastic molecular events can be successfully inhibited by BAY 11-7082, a specific pharmacologic NF-κB inhibitor, further supporting the role of NF-κB as a key mediator of bile-induced oncogenic events in the laryngopharyngeal epithelium [18,19,20,21,22].

Constitutive activation of NF-κB is observed in various cancers, including HNSCC [23,24]. It has been shown that HNSCC exhibit abundant NF-κB activation, and several studies indicate that NF-κB is upregulated in premalignant lesions and invasive cancers [25,26,27,28]. The importance of NF-κB in the initiation and progression of cancer, including head and neck malignancies, has been widely supported by its interactions with a complex network of other cancer-related transcriptional factors, cytokines, and growth factors. These commonly include EGFR/Ras/RAF/MAPK, Akt/PI3K/mTOR, ΙΚΚ/NF-κB, STAT3, and wnt/β-catenin [25,26,29]. 

MicroRNA (miRNA) molecules have additionally been considered to play an important role in both inflammation and cancer [28], while some miRNAs, such as “oncomirs” and “tumor suppressor” miRNAs, are post-transcriptionally capable of contributing to carcinogenesis via their regulatory role in the multistep process of cancer initiation and progression [30,31]. Previous studies have demonstrated that the deregulation of “oncomirs” *miR-21*, *miR-155*, and *miR-192*, and tumor suppressors *miR-375*, *miR-451a*, and *miR-34a* are associated with laryngopharyngeal cancer [32,33,34,35,36]. An independent association has been demonstrated between NF-κB activation and the upregulation of oncogenic *miR-21* and/or downregulation of tumor suppressor *miR-34a* and *miR-451a* [18,20,37,38,39], while a cluster of genes and miRNA markers have been implicated in activating NF-κB and contributing to an aggressive phenotype of head and neck cancer [39]. 

To suggest that bile may be etiologically implicated in supraesophageal squamous cell carcinoma (SCC) in humans, we have recently shown that unlike tumors of patients without biliary esophageal reflux, NF-κB is highly activated in patients with bile-related hypopharyngeal SCC, accompanied by characteristic premalignant mRNA and miRNA phenotypes [40] that we had previously identified in acidic-bile-treated murine hypopharyngeal mucosa (HM) [16,17].

Therefore, to buttress the role of acidic bile reflux, we extended the exposure in the in vivo mouse model to elucidate whether the lesions of dysplasia progressed to fully invasive carcinoma under continued intermittent exposure to acidic bile.

## 2. Results

### 2.1. Long-Term Exposure of HM to Acidic Bile Progressively Induced a Malignant Histopathologic Phenotype 

**Figure 1:**
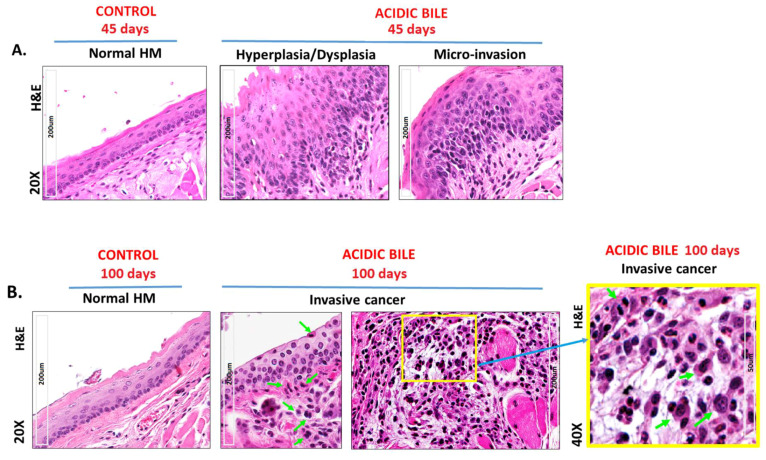
Acidic-bile-induced malignant transformation in the murine hypopharyngeal mucosa (HM) of C57Bl/6J mice (H&E staining) after 45 days and 100 days of exposure (from left to right). (**A**) The 45 days of exposure. Normal HM—keratinized stratified squamous epithelium/single layer of basal cells; hyperplastic/dysplastic HM—the thickness of the stratified epithelium and hyperchromatic or pleiomorphic basal cells extended into the upper layers of the mucosa with mitotic figures seen throughout the depth of mucosa; severe dysplasia/micro-invasion—architectural changes extended into the upper levels of the mucosa, with mitotic features evident throughout the depth of the mucosa. Submucosal invasion by basal cells while maintaining full-thickness nuclear hyperchromatism without surface maturation. (**B**) The 100 days of exposure. Normal HM—keratinized stratified squamous epithelium/single layer of the basal cells; invasive carcinoma—a representative malignant phenotype of atypical neoplastic cells in the submucosa characterized by large oval nuclei with abundant mitotic figures (arrows).

Microscopic examination of acidic-bile-exposed HM demonstrated both pre-malignant changes, including hyperplasia, dysplasia, and micro-invasion (45-day exposure) (Supplementary data 2.1) (Figure 1A); as well as the invasive malignant phenotype (100-day exposure) depicted in Figure 1B and Table S1.

**Figure 2:**
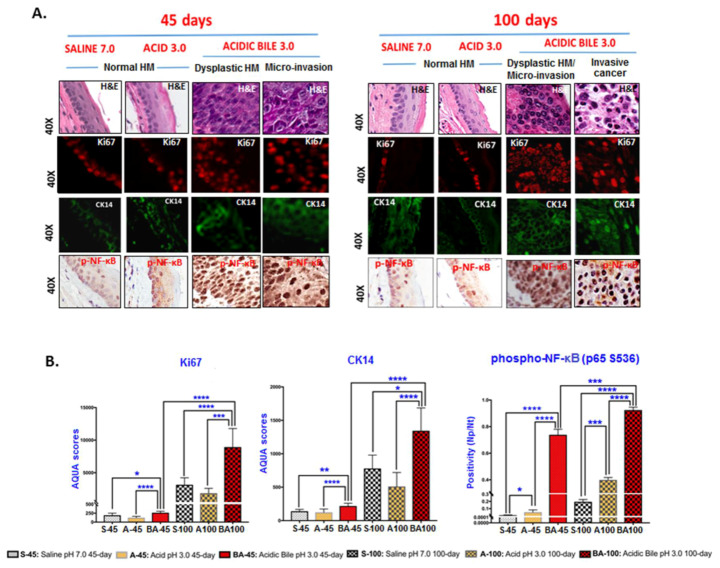
Underlying molecular alterations linked to histopathological changes at 45 days and 100 days of exposure. (**A**) Immunofluorescence staining was used for Ki67 (DyLight^®^549 for red) and CK14 (DyLight^®^488 for green) (DAPI was used for nuclear staining (not seen here)). IHC analysis for p-NF-κB (p65 S536) (brown) was performed using chromogenic staining. (**B**) Graphs created by GraphPad Prism 7.0 demonstrate the statistically significant differences of mean AQUA (automated quantitative analysis) scores for Ki67 (nuclear) and CK14 (nuclear and cytoplasmic) or nuclear positivity for p-NF-κB (positivity = nuclear − positive/total number of nuclei; Image Scope software) between acidic bile vs. controls, and between acidic bile 45-day vs. acidic bile 100-day treated groups (*p < 0.01; **p < 0.001; ***p < 0.0001; ****p < 0.00001; *t*-test; multiple comparisons using Holm–Sidak; GraphPad Prism 7.0). The BA 100 in the bar graphs represent both dysplastic HM and invasive cancer.

**Figure 3:**
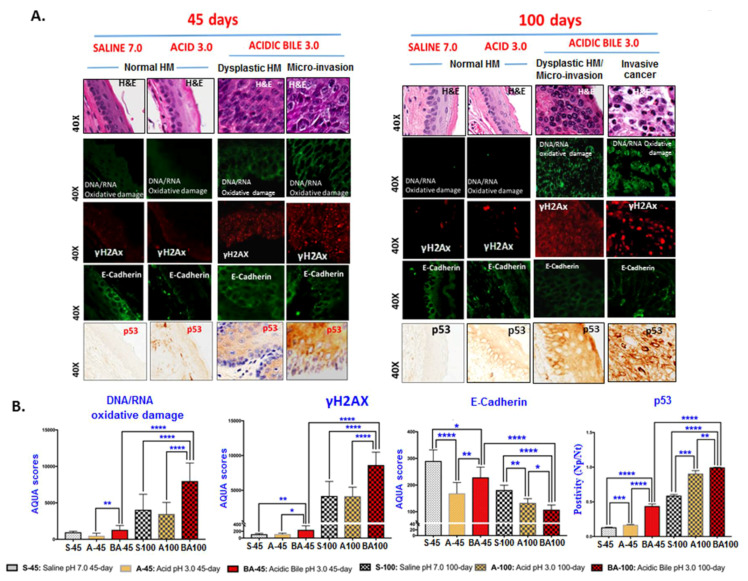
Acidic-bile-induced DNA damage and increased expression of p53 at 45 days and 100 days of exposure. (**A**) Immunofluorescence staining was used for DNA/RNA oxidative damage markers (green), γH2AΧ (p-H2AX (S139)) (DyLight^®^549 red) and E-Cadherin (DyLight^®^488 for green) (DAPI was used for nuclear staining (not seen here)). IHC analysis for p53 (brown) was performed using chromogenic staining. (**B**) Graphs created using GraphPad Prism 7.0 demonstrate the statistically significant differences of the mean AQUA (automated quantitative analysis) scores for DNA/RNA oxidative damage markers (nuclear and cytoplasmic), γH2AΧ (nuclear), and E-Cadherin (membrane/cytoplasmic) or cytoplasmic positivity for p53 (positivity = positive p53 staining/total number of cells; Image Scope software) between acidic bile vs. controls, and between acidic bile 45-day vs. acidic bile 100-day treated groups (* *p* < 0.01; ** *p* < 0.001; *** *p* < 0.0001; **** *p* < 0.00001; *t*-test; multiple comparisons using Holm–Sidak; GraphPad Prism 7.0). The BA 100 in the bar graphs represents both dysplastic HM and invasive cancer.

Figure 1B displays markedly atypical nested cells in the submucosa characterized by large oval nuclei with abundant mitotic figures. IHC analysis using cell proliferation marker Ki67 superimposed with a specific marker for basal layer keratinocytes CK14 [41] confirmed the presence of invasive proliferating epithelial cells (Figure 2A). IHC staining for DNA/RNA oxidative damage and γH2AX, a marker of DNA double-strand breaks (DSBs), was evidence for DNA damage in the malignant cell population (Figure 3A; Figure S1). 

All controls demonstrated a weak or negative staining for the cell proliferation marker Ki67 (Figure 2A). Staining for CK14 was limited to the basal layer of the normal epithelium control (Figure 2A). Controls using saline at pH 7.0 and acid (pH 3.0) alone were also found to be negative for DNA/RNA oxidative damage and γH2AX (Figure 3A; Figure S1). Acid-alone-treated HM demonstrated a weak staining for E-Cadherin compared to saline-treated HM at pH 7.0, where we noted an intense E-Cadherin staining through its full thickness (Figure 3A). 

Automated quantitative analysis (AQUA) revealed progressive changes of the expression levels in cell proliferation (Ki67, CK14), DNA damage (γH2AX and DNA/RNA oxidative damage), and cell-cell adhesion marker E-Cadherin compared to the controls (acidic-bile-treated vs. acid-alone- or saline-treated HM, *p* < 0.005 and *p* < 0.05, respectively) (Figure 2B; Figure 3B). It was noteworthy that acidic-bile-exposed HM for 100 days demonstrated the highest Ki67, CK14, γH2AX, and DNA/RNA oxidative damage markers levels, and the lowest E-Cadherin levels, among the analyzed groups (100 days vs. 45 days of acidic-bile-treated HM, *p* < 0.0001, *t*-test; means ± SD; multiple comparisons using Holm–Sidak) (Figure 2B; Figure 3B), supporting the observation that chronic exposure of HM to bile at acidic pH (3.0) accelerated the DNA damage and changes in proliferation markers, as well as decreased the cell–cell interactions. 

### 2.2. Long-Term Exposure to Acidic Bile Progressively Induced Increased NF-κB Activation and Elevated Expression Levels of p53 in HM

The microscopic examination of acidic-bile-treated HM, particularly at sites of pre-malignant and malignant changes, revealed a significant activation of NF-κB (p65 S536) and significantly higher p53 levels relative to normal HM (saline and acid-alone controls) using IHC analysis (Figure 2A; Figure 3A). 

We found that although acidic bile produced an intense p-NF-κB nuclear staining of cells in basal and suprabasal layers in the first 45 days of treatment, 100 days of long-term exposure to acidic bile induced even higher levels of activated NF-κB, with a significant difference compared to the controls (*p* < 0.0005, *t*-test; means ± SD; multiple comparisons using Holm–Sidak) (Figure 2B). Acid alone induced less intense and less extensive NF-κB activation in HM limited to the basal layer compared to bile-treated HM. 

We also found that acidic bile produced an intense p53 staining of cells in exposed mucosa and cells invading the submucosa. Although HM treated with acidic bile for 45 days induced higher p53 levels compared to the controls (*p* < 0.005), 100 days of exposure to acidic bile induced significantly higher levels of p53 compared to the 45 days samples (*p* < 0.0001, *t*-test; means ± SD; multiple comparisons using Holm–Sidak) (Figure 3B). Saline and acid controls were found to be negative for p53 staining, whereas HM with long-term exposure to acid alone showed a weak reactivity for p53.

### 2.3. Correlation Analysis

A Spearman nonparametric test demonstrated strong linear correlations (*p* < 0.05) among the analyzed NF-κB (phospho-p65 S536), p53, cell proliferation markers (Ki67 and CK14), cell-adhesion molecules (E-Cadherin), and DNA damage markers (DNA/RNA oxidative markers and γH2AX marker for DNA DSBs) (Supplementary data 2.2). 

### 2.4. Long-Term Exposure of HM to Acidic Bile Progressively Induced Increased Transcriptional Levels of an Oncogenic Phenotype

**Figure 4:**
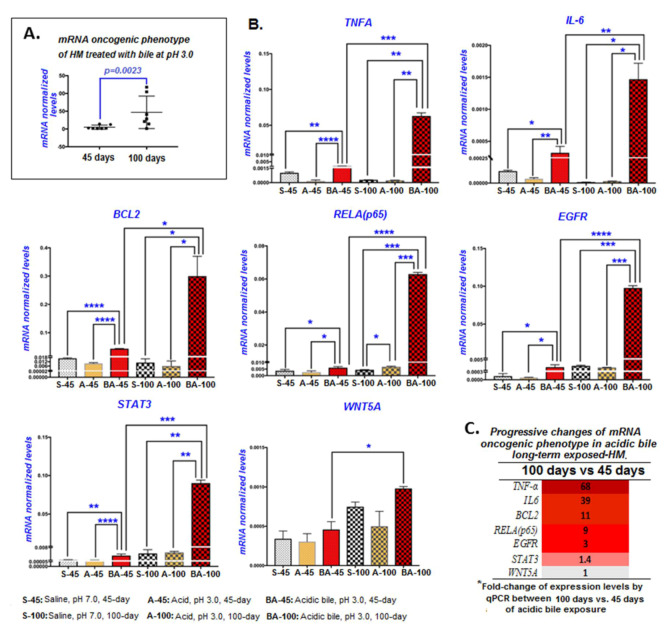
Progressive transcriptional activation of the NF-κB related oncogenic phenotype. (**A**) mRNA levels of all the analyzed NF-κB-related genes (*Bcl2*, *Rela*, *Egfr*, *Wnt5a*, *Stat3*, *Tnf*, and *Il6)* (mRNA levels of genes normalized to *Gapdh*; real-time qPCR analysis) after 45 and 100 days of exposure to acidic bile (pH 3.0) vs. the saline control. Statistical analysis reveals significantly higher mRNA ratios of the analyzed genes at 100 days vs. 45 days (*p*-value using one-way ANOVA; Friedman test). (**B**) Transcriptional levels of each analyzed gene in HM exposed for 45 and 100 days to saline (pH 7.0), acid alone (pH 3.0), and acidic bile (pH 3.0) (mRNA levels of each target gene were normalized to *Gapdh*; real-time qPCR analysis; Graph Pad Prism software 7.0). We observed significantly higher mRNAs in the HM exposed to acidic bile for 100 days vs. 45 days (* *p* < 0.01; ** *p* < 0.001; *** *p* < 0.0001; **** *p* < 0.00001; *t*-test; multiple comparisons using Holm–Sidak; GraphPad Prism 7.0; data obtained from four analyzed samples). (**C**) Progressive changes of the mRNA oncogenic phenotype (fold-change of mRNAs) at 100 days relative to 45 days.

The long-term (100 days) exposure of HM to acidic bile progressively increased the transcriptional activation of the mRNA oncogenic phenotype compared with the 45 days of exposure (Figure 4A). Specifically, *Tnf*, *Il6*, *Egfr*, *Bcl2*, *Stat3*, *Wnt5a*, and *Rela* mRNAs were highly activated in HM (Figure 4B; Table S4), producing significantly higher mRNA relative expression changes (acidic-bile-treated vs. saline-treated HM) after 100 days compared to the first 45 days of exposure (Figure 4C). 

In general, the long-term exposure of HM to acidic bile induced higher transcriptional levels of NF-*κ*B transcriptional factor *Rela,* cancer-related inflammatory molecules *Tnf* and *Il6*, anti-apoptotic gene *Bcl2*, and oncogenic *Egfr* and *Stat3*, with statistically significant differences compared to HM exposed to acid alone (pH 3.0), or saline (pH 7.0) treated controls (Figure 4B). The exposure of HM to acid alone for 45 or 100 days induced lower mRNA levels of the oncogenic profile compared to the saline-treated controls. However, HM treated for acid alone for 100 days displayed significantly higher mRNA levels of *Rela* compared to saline-treated HM, suggesting that long-term exposure to strongly acidic pH contributes to NF-κB overexpression (Figure 4B).

### 2.5. Long-Term Exposure of HM to Acidic Bile Progressively Induced Increased Deregulations of Inflammatory-Related miR-155 and the Tumor Suppressors miR-375 and miR-451a

MicroRNA analysis demonstrated that 100 days of chronic exposure of HM to bile at a strongly acidic pH (3.0) induced deregulations of the miRNA oncogenic phenotype, consisting of the upregulated “oncomirs” *miR-21*, *miR-155*, and *miR-192*, and the downregulated “tumor suppressors” *miR-34a*, *miR-375*, and *miR-451a*, which is consistent with our prior findings from in vitro and in vivo explorations using short-term exposures of acidic bile on murine laryngopharyngeal mucosa [17,18,20,22]. 

**Figure 5:**
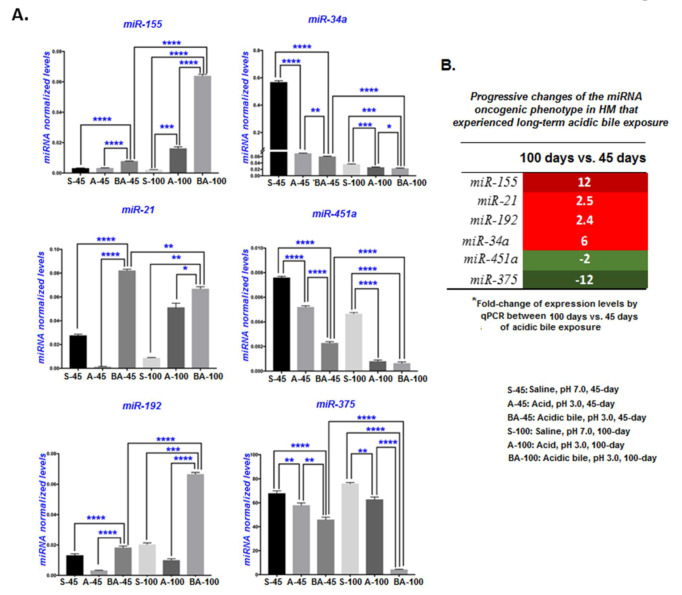
Progressive deregulation of the miRNA oncogenic phenotype. (**A**) Expression levels for each analyzed miRNA marker at 45 days and 100 days of exposure to saline (pH 7.0), acid alone (pH 3.0), and acidic bile (pH 3.0) (miRNA levels of each marker were normalized to *RNU6*; real-time qPCR analysis; Graph Pad Prism software 7.0). We observed a significant upregulation of “oncomirs” *miR-21*, *miR-155*, and *miR-192*, and a significant downregulation of “tumor suppressors” *miR-375* and *miR-451a* in HM exposed to acidic bile for 100 days vs. 45 days (* *p* < 0.01; ** *p* < 0.001; *** *p* < 0.0001; **** *p* < 0.00001; *t*-test; multiple comparisons using Holm–Sidak; GraphPad Prism 7.0; data obtained from four analyzed samples). (**B)** Progressive changes of the miRNA oncogenic phenotype (fold-changes of miRNAs) in the HM expose to acidic bile for 100 days vs. 45 days.

Long-term (100 days) acidic bile exposure induced a progressive and significant upregulation of “oncomirs” *miR-21*, *miR-155*, and *miR-192* compared to controls, and significant upregulation of *miR-155* and *miR-192* compared to 45 days of acidic bile exposure (Figure 5A; Table S5). The table in Figure 5B presents the relative expression changes (acidic-bile-treated vs. saline-treated HM) of the analyzed “oncomirs” between 45 and 100 days of exposure, showing a progressive upregulation of the inflammatory-related *miR-155* (12-fold changes) over time. Acid alone also produced significantly higher levels of *miR-155* compared to the saline-treated controls, supporting an observation that *miR-155* expression can be upregulated by strongly acidic pH (Figure 5A). 

The long-term (100-day) acidic bile exposure also induced a significant downregulation of tumor suppressors *miR-375*, *miR-34a*, and *miR-451a* compared to controls, and produced a progressive downregulation of *miR-375* (12-fold changes) and *miR-451a* (2-fold changes) compared to the 45-day acidic bile exposure (Figure 5B; Table S5). Although the “tumor suppressor” *miR-34a* was also significantly downregulated in the HM exposed to acidic bile for 100 days (Figure 5A; Table S5), a more profound effect was found in the first 45 days relative to an extended time of 100 days of exposure (Figure 5B; Table S5).

A Spearman non-parametric test revealed statistically significant linear correlations (*p* < 0.05) between the acidic bile-induced mRNAs of NF-κB related genes and miRNA markers supporting interactions among the NF-κB-related phenotypes [18,20] (Supplementary data 2.2). 

## 3. Discussion

**Figure 6:**
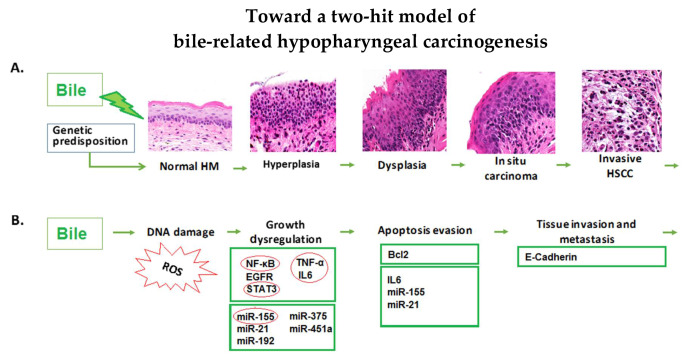
Bile and hypopharyngeal carcinogenesis. (**A**) In vivo model supporting the histologic progression in hypopharyngeal mucosa (HM) under the carcinogenic effect of acidic bile refluxate. (**B**) Genetic alterations in HM caused by chronic bile exposure contribute to hypopharyngeal carcinogenesis. Biliary acidic refluxate causes oxidative damage resulting in high levels of ROS or double-strand breaks that can incur direct DNA damage. Direct DNA damage has been shown to lead to tumor-initiating mutations in head and neck cancer [42,43]. ROS can also activate several cancer-associated signaling pathways including NF-κB [42]. Chronic exposure to acidic bile induces a systematic release of cytokines, such as IL-6 and TNF-α that are involved in oncogenic pathways [27]. Long-term exposure to acidic bile also induces a significant overexpression of *Bcl2*, further supporting the anti-apoptotic effect of chronic acidic bile refluxate in hypopharyngeal mucosa [27]. Acidic bile exposure of HM is also capable of directly inducing significant deregulation of cancer-related miRNAs, which are known regulatory molecules that mediate inflammation and tumorigenesis [31]. In particular, chronic intermittent exposure of HM to bile at a strongly acidic pH induces a profound upregulation of *miR-155*, supporting a possible chronic inflammatory effect associated with neoplastic events [30]. Long-term exposure to acidic bile induces a significant decrease of E-Cadherin consistent with progressive invasion. The combination of direct DNA damage and the NF-κB-related anti-apoptotic deregulation provide early evidence for the two-hit model of carcinogenesis.

Although several epidemiologic studies have come to support the role of LPR in neoplasia of the upper aerodigestive tract [4,5,10], the precise role of bile-containing laryngopharyngeal refluxate in supraesophageal cancers and the underlying mechanism of its carcinogenic effect so far remains unresolved. Our novel findings demonstrate that chronic intermittent exposure of hypopharyngeal mucosa to acidic bile is capable of producing a progressive malignant transformation (Figure 6). The mechanistic role of NF-κB, shown to appear as early as 7 days [18], in the biliary-related neoplastic process is also supported by the identified progressive deregulations of its related oncogenic mRNA and miRNA phenotypes that precede the histologic evidence.

Using an in vivo model, we provide evidence that the malignant transformation of hypopharyngeal mucosa under the effect of acidic bile is a progressive process that depends on the duration of exposure. Specifically, our novel findings document that repetitive exposure to acidic bile produced pre-malignant changes that gradually progressed to malignancy, as evidenced by the earlier effect of acidic bile in inducing premalignant changes, such as hyperplasia and mild-to-moderate dysplasia, in the 45 days of exposure [16]. Here, we further document for the first time that exposure to acidic bile for 45 days was capable of producing a micro-invasion of the basal mucosal layer, accompanied by DNA/RNA oxidative damage and double-strand breaks (DSBs) (increased positivity for γH2AX marker) that are linked to tumor-initiating mutations in head and neck cancers [42,43] (Figure 6). 

Although micro-invasion is considered to be an early step toward malignancy, our novel findings demonstrate that continued exposure to acidic bile promoted invasion of the underlying stroma at 100 days. We confirmed that the atypical invading cells proved to be proliferating epithelial cells (positive for Ki67 and CK14) characterized by a profound positivity for the DNA DSBs damage marker, an indicator of the mutagenic process. Our findings from immunohistochemical analysis also demonstrated significantly lower levels of E-Cadherin in the 100 days of mucosal exposure compared to 45 days and the controls, which is consistent with progressive invasion.

We further observed the elevation of p53 expression in both pre-malignant lesions (45-day exposure) and malignant sites after 100 days of exposure. *Trp53*, a tumor suppressor gene that regulates target genes of the cell cycle [44], is usually expressed at low protein levels in normal cells and often at high levels in varieties of transformed cell lines, where it is believed to contribute to malignancy [44]. Specifically, it has been previously reported that quantitative increases in the expression of either mutant and/or wild-type p53 protein are biologically significant in the neoplastic process of tumors [45]. Our data from IHC, using a p53 antibody that reacts with either mutant as well as its wild form, shows an acidic-bile-induced progressive increase of overall p53 protein, supporting its contribution in a bile-reflux-related neoplastic process.

Our novel findings show that the progressively induced histopathological changes were preceded by the gradually increasing transcriptional activation of cancer-related factors and deregulations of miRNA molecules. In the first 45 days of acidic bile exposure, the HM produced a significant overexpression of the NF-κB-related mRNA oncogenic phenotype, including *Rela*, *Stat3*, *Il6*, *Egfr*, *Tnf*, *Bcl2*, and *Wnt5a*, compared to controls, which is consistent with our prior observations [16]. After 100 days of exposure, there appeared to be a significantly higher overexpression of cancer-related cytokines *Tnf* and *Il6*, anti-apoptotic *Bcl2*, NF-κB factor *Rela*, and oncogenic factor *Egfr* compared to the 45-day exposure (Figure 4B,C). These observations are of interest because they support underlying tumorigenesis that is progressive (Figure 6).

The above observation was further supported by a consideration of the identified miRNA oncogenic phenotypes in the first 45 days, compared to the total 100-day period of exposure. Consistent with our prior published data, in the first 45 days of acidic bile exposure, the expression of “oncomirs” *miR-21*, *miR-155*, and *miR-192*, and the “tumor suppressors” *miR-34a*, *miR-451a*, and *miR-375* were significantly deregulated [17]. After 100 days of exposure, the miRNA oncogenic phenotype demonstrated a significantly higher overexpression of the “oncomirs” *miR-21* (2.5-fold), *miR-192* (2.5-fold), and particularly *miR-155* (12-fold) compared to the 45-day exposure (Figure 5B). Additionally, we observed a greater downregulation of “tumor suppressors” *miR-451a* (2-fold) and *miR-375* (12-fold) within a similar window of comparison (Figure 5B). 

Even a short 7-day exposure to acidic bile can induce early NF-κB activation and pre-neoplastic molecular events that are capable of being inhibited by the topical application of NF-κB inhibitor BAY 11-7082, confirming its role as a key mediator of early bile-induced pre-neoplastic events [18]. The use of this inhibitor also provides important opportunities to prevent pre-oncogenic properties of biliary esophageal reflux, encouraging further exploration that may exert clinical impacts.

Finally, two opportunities unaddressed in this report require comment. First, although prior studies have shown that zinc-finger proteins, such as poly(ADP-ribose) polymerase or PARP-1 [46], may further support the presence of DNA damage, its role has been difficult to clearly define or categorize. PARP enzymes seem to play a wide variety of roles ranging from DNA repair to the regulation of gene expression and cell death [46]. On the one hand, there is evidence that PARP may be an important cofactor in the activation of NF-κB-dependent target genes [47,48]. On the other hand, the absence of PARP-1 did not affect their activation [49]. While our current analysis did not include PARP, it did include markers of DNA damage for the detection of four enzymes (γH2Ax (p-H2AX (S139)) for DNA DSBs and a cocktail of oxidative DNA/RNA damage antibodies with a high specificity and affinity for oxo8dG (8-hydroxy-2′-deoxyguanosine), oxo8Gua (8-oxo-7,8-dihydroguanine), and oxo8G (8-oxo-7,8-dihydroguanosine)) that are capable of documenting high levels of acidic bile-induced DNA damage (Figure 3). Nevertheless, the role of PARP in activating NF-κB target genes is an important potential relationship to pursue, providing further exploratory opportunity for the support of a two-hit model. 

Second, although several nuclear receptors (NRs) have been identified in the head and neck [50], their association with bile acids has not yet been described. In contrast, in the gastrointestinal tract, interactions have been described between certain bile acids and membrane receptors, such as Takeda G-protein coupled receptor (TGR5) [51]. Bile acids are also found to activate nuclear farnesoid X receptors (FXRs) in the lower esophagus [52,53] that contribute to precancerous lesions [54]. We believe that the identification of specific receptors activated by acidic bile is a crucial area for future research, presenting a wide range of opportunities for potential therapeutic intervention. We, therefore, believe the role of FXR and TGR5 deserves further exploration in the hypopharynx.

## 4. Materials and methods

### 4.1. In Vivo Model 

Using *Mus musculus*, mouse strain C57Bl/6J (Jax mice, Jackson Laboratory USA) (24 males and 24 females; 8 mice (8 males + 8 females) per group), we performed repeated topical applications on murine hypopharyngeal mucosa (HM), two times per day for 45 and 100 days (Table S1), using a mixture of conjugated bile salts (10 mmol/L in buffered saline) (≈4 μmol per day) at molar concentrations previously described and considered to be close to those measured in aspirates from patients [7,15,55], at an acidic pH 3.0, adjusted using 1M HCl (Supplementary methods 1.1). 

The experimental and control groups included (i) bile at acidic pH 3.0, (ii) acid alone without bile salts (buffer saline; pH 3.0), and (iii) a saline-treated control group at pH 7.0. Procedures followed the approved protocol 11039 of IACUC (Yale University; New Haven, CT, USA).

### 4.2. Tissue Examination 

The histologic staining of the HM tissue sections using hematoxylin and eosin (H&E), along with the tissue examination, is described in Supplementary methods 1.2. Histopathological alterations were assessed according to previously described criteria (WHO, Ljubljanska [56,57] and laboratory mouse histology [58]). 

### 4.3. Immunohistochemical, Gene Expression, and miRNA Analysis

We performed immunohistochemical (IHC) analysis for Ki67, cytokeratin 14, E-Cadherin, γH2AX [p-H2AX (S139)], DNA/RNA oxidative damage markers, p-NF-κB (p65 S536), and p53 proteins, as described in Supplementary methods 1.3. The protein expression was measured using a PM-2000 image workstation and HistoRX^®^ software (HistoRx Inc., New Haven, CT, USA) (automated quantitative analysis or AQUA), or Image Scope software (Leica Microsystems, Buffalo Grove, IL, USA), as described previously [16]. 

We also performed quantitative real-time PCR analysis to determine the transcriptional levels of *Rela*, *Egfr*, *Tnf*, *Bcl2*, *Il6*, *Wnt5a* (*Gapdh* was used as a reference control gene), and “oncomirs” *miR-21*, *miR-155*, *miR-192*, and “tumor suppressors” *miR-34a*, *miR-375*, and *miR-451a* (*RNU6B* reference control), previously linked to laryngopharyngeal cancer [16,17,18,19,21,22,32,33,34,35,36,37,38,39,40,59,60] (Supplementary methods 1.4 and 1.5; Tables S2 and S3). Statistical analysis was performed using GraphPad Prism 7.0 software (GraphPad Software Inc., San Diego, CA, USA), as described in Supplementary methods 1.6. 

## 5. Conclusions

Our in vivo findings provide novel evidence that bile in the upper aerodigestive tract is pathogenic and worthy of our consideration as a risk factor in the development of hypopharyngeal squamous cell cancer. We provide evidence that the progressive phenotype is strongly supported and preceded by a specific and characteristic NF-κB-related oncogenic profile (Figure 6). This is the first study to document that acidic bile is carcinogenic in the upper aerodigestive tract. As such, these findings encourage us to further characterize thοse molecular events and mechanisms that may be signatures of the recognition of a new etiopathogenesis. Although other clinical risk factors, such as smoking and alcohol, as well as gastric (acid) reflux, may affect the hypopharyngeal mucosa [3,5], identifying and characterizing the role of gastroduodenal fluid as an independent causal risk factor in head and neck malignancies would be considered novel and would eventually allow for risk stratification and prevention of laryngopharyngeal cancer or its recurrences, including the development of second primaries in high-risk patients. Equally as important, we reproduced the role of several biomarkers of progression that can serve as valuable indicators of early neoplasia in our experimental model. These findings provide a sound basis for proposing translational studies in humans by exposing new opportunities for early detection and prevention.

## Supplementary Materials

The following are available online at https://www.mdpi.com/2072-6694/12/5/1064/s1, Supplementary methods and data, Table S1: Percentage (%) of C57BL/6J mice exhibiting histopathological alterations of hypopharyngeal mucosa (HM) after 45 and 100 days of treatment; Table S2: Mouse genes (target and reference Gapdh) and their detected transcripts, analyzed using real-time qPCR, in murine HM; Table S3: Mouse mature miRNAs (targets) and reference RNU6-2 small RNA control, analyzed using real-time qPCR, in murine HM; Table S4: (A) The 45-day and 100-day acidic-bile-induced transcriptional levels of the NF-κB-related oncogenic pathway in murine HM. (B) Relative mRNA expression ratios for each target gene in murine HM exposed to acidic bile for 45 and 100 days relative to the corresponding saline-treated controls; Table S5: (A) The 45-day and 100-day acidic-bile-induced miRNA levels in exposed murine HM. (B) Relative miRNA expression ratios for each marker in murine HM exposed to acidic bile for 45 days and 100 days relative to the corresponding saline-treated controls), and Figure S1: Acidic bile-induced chromogenic immunohistochemical staining for DNA damage at 45 days and 100 days of exposure.
