# Supplementary material for: The Progressive Mutagenic Effects of Acidic Bile Refluxate in Hypopharyngeal Squamous Cell Carcinogenesis: New Insights
Source: Cancers (Basel). 2020 Apr 25;12(5):1064. doi: 10.3390/cancers12051064 (PMC7281001; doi:10.3390/cancers12051064)
Supplement: Supplementary file 1 [file cancers-12-01064-s001.pdf]

Supplementary Material: The Progressive Mutagenic Effects of Acidic Bile Refluxate in Hypopharyngeal Squamous Cell Carcinogenesis: New Insights

Clarence T. Sasaki, Sotirios G. Doukas, Jose Costa and Dimitra P. Vageli

1. Supplementary Methods

1.1. In Vivo Model

Using *Mus musculus*, mouse strain C57Bl/6J (Jax mice, Jackson Laboratory USA) (24 males and 24 females; 8 mice (8 males + 8 females) per group), we performed repeated topical applications on murine hypopharyngeal mucosa (HM), two times per day for 45 and 100 days (Table 1), using a mixture of conjugated bile salts (10 mmol/L in buffered saline) (≈4 μmol per day) at molar concentrations previously described and considered to be close to those measured in aspirates from patients [7,15,55] at acidic pH 3.0, adjusted using 1M HCl. Although we have previously shown the dysplastic effects of acidic bile on HM at 45 days [16] we repeated the 45 days exposure here to support a comparison at 100 days under identical conditions that would highlight both molecular and histologic progression to malignancy. The experimental and control groups included (i) bile at acidic pH 3.0, (ii) acid alone without bile salts (buffer saline; pH 3.0), and (iii) a saline-treated control group at pH 7.0 (reference control for the mechanical effect of the feeding tube on HM). Procedures followed the approved protocol 11039 of IACUC (Yale University, New Haven, CT, USA). At 45 days, half of the experimental and control animals were euthanatized, while the remaining subjects were euthanatized at 100 days (according to IACUC euthanasia policy and guidelines). The euthanized animals were kept on ice for the dissection of HM tissue fragments. The HM from four animals (two males and two females) of each group, at 45 and 100 days, were immediately placed into 10% neutral buffered formalin (Thermo Fisher Scientific, Middletown, VA, USA) to be submitted for embedding in paraffin blocks (Yale Pathology Facilities), while the remaining tissue fragments from each experimental and control groups were immersed in an RNA stabilization solution (RNAlater, Life Technologies, Grand Island, NY, USA) and kept at −80°C for RNA isolation.

1.2. Tissue Examination-Histology

Histological staining: We examined hematoxylin and eosin (H&E)-stained 3 μm thick tissue sections of that were formalin-fixed and paraffin-embedded (FFPE), using light microscopy. Images were captured and analyzed using Aperio CS2, Image Scope software (Leica microsystems, IL, USA). Histopathological alterations were assessed according to criteria, as previously described [56,57] and laboratory mouse histology [58].

Controls: Stratified keratinizing squamous epithelium with a single layer of basal cells characterized normal HM served as controls.

Pre-malignant changes: Abnormal hyperplasia was characterized by thickened stratified epithelia with an expansion of basal cells into the suprabasal layer without cytologic atypia, considered by some as a pre-cursor for malignant transformation [56,57]. In the case of a dysplastic epithelium, architectural disorder and hyperchromatic or pleomorphic basal cells were found to expand in the stratum spinosum. Moderate dysplasia was characterized by nuclear hyperchromatism with a high degree of basal layer expansion and/or nuclear hyperchromatism with an increase of nuclear to cytoplasm ratios, and/or a loss of cell polarity into the middle third of the mucosa. Severe dysplasia characterized by similar architectural changes extended into the upper levels of the mucosa with mitotic figures evident throughout the depth of the mucosa. Micro-invasion was characterized by submucosal invasion of basal cells from dysplastic HM.

Malignant transformation: Malignant transformation was characterized by atypical squamous cells with abundant pink keratinizing cytoplasm invading the submucosa with hyperchromatic

nuclei and mitotic figures, the presence of intercellular bridges of atypical cells, and hypercellularity. (all tissue specimens were examined to exclude histological signs of local treatment toxicity, such as hemorrhagic lesions, ulceration, or inflammation).

1.3. Immunohistochemical Analysis

Immunofluorescence (IF) staining: We selected at least two tissue specimens from each experimental and control group, including specimens with pre-malignant/malignant lesions, to identify molecular alterations related to bile-induced malignant transformation of HM and DNA damage. IF staining was performed in serial mucosal sections of the selected specimens for (i) cell proliferation marker Ki67 (anti-Ki67, rabbit mAb, SP6, Thermo Scientific™ Lab Vision, Walthamn MA, USA); (ii) cytokeratin 14 specific for basal layer epithelial cells (anti-cytokeratin 14; #ab7800, mouse mAb, LL002, Abcam®, Cambridge, UK); and (iii) E-Cadherin, a cell–cell adhesion molecule (anti-E-Cadherin, mouse mAb, NCH-38, Dako, Carpinteria, CA, USA). We also used (iv) anti-γH2Ax (Phospho-Histone H2A.X (Ser139/Tyr142); Cell signaling, Danvers, MA, USA) to detect DNA DSBs in dysplastic basal/suprabasal cells and in examples of histopathologic invasion, and (v) anti-DNA/RNA oxidative damage antibody (Anti-DNA/RNA Damage antibody, clone 15A3, Abcam) with a high specificity and affinity to oxo8dG (8-hydroxy-2'-deoxyguanosine), oxo8Gua (8-oxo-7,8-dihydroguanine), and oxo8G (8-oxo-7,8-dihydroguanosine), to detect oxidative DNA damage in dysplastic layers and the foci of invasion.

We used anti-rabbit or anti-mouse secondary DyLight®488 for CK14, E-cadherin and DNA/RNA oxidative damage markers (green), DyLight®549 for Ki67 and γH2Ax (red), and DAPI (blue) to distinguish the nuclei (DyLight®488 and DyLight®549; Vector Labs, Burlingame, CA, USA). At the end of the IF staining, the slides were examined microscopically and their images were captured for analysis (Zeiss fluorescence microscope, AxionVision system; Carl Zeiss microscopy, White Plains, NY, USA).

Automated quantitative analysis (AQUA): AQUA analysis was used to measure the protein expression levels of Ki67, CK14, E-cadherin, γH2Ax, and DNA/RNA oxidative damage markers in bile-treated HM relative to the controls. In brief, images of tissue sections (derived from each experimental and control group of animals) were captured and analyzed using the PM-2000 image workstation and HistoRX® software (HistoRX Inc., New Haven, CT 06511, USA), as previously described [16,17]. For each whole-tissue section, areas of dysplastic HM and the foci of invasion were selected, while the remaining compartments of submucosa were excluded, using AQUA software. The signal intensity of the target antigen was acquired using DyLight®488 (similar to FITC) signal or DYLight®549 (similar to Cy3) signal, relative to the tumor mask. The signal intensity of nuclei was acquired using a DAPI signal. AQUA scores within the nucleus and cytoplasm (or membrane) were calculated by dividing the signal intensity by the area of the specified compartment.

Chromogenic immunohistochemical analysis: Chromogenic immunohistochemical (IHC) analysis was performed using immunoperoxidase (DAB peroxidase substrate), for p-NF-κB and p53, in tissue sections from all experimental and control specimens to identify NF-κB activation and p53 overexpression in bile-exposed murine HM compared to controls. We used 1:100 dilutions of anti-phospho-p65 (rabbit polyclonal anti-phospho-p65 Ser536, AbD Serotec, Bio-Rad, Hercules, CA, USA), as previously described [16]. We also used p53 antibody (anti-p53, Ab-6, clone DO-1, cat # MS-187; Thermo-Scientific) that reacts with mutant p53, as well as its wild form (p-NF-κB and p53: brown). We also performed chromogenic IHC analysis for anti-γH2Ax (Cell signaling; Danvers, MA, USA) and Anti-DNA/RNA Damage antibody (clone 15A3, Abcam®, Cambridge, UK) (brown) to confirm DNA damage to the nuclear and cytoplasmic compartments of epithelial cells from IF staining data.

We used positive controls and a non-template negative control in each IHC assay, as recommended by the manufacturer. Slides were analyzed using a Leica light microscope and images were captured using an Aperio CS2. The images were analyzed using Image Scope software (Leica Microsystems, Buffalo Grove, IL, USA) that generated algorithm(s) illustrating the mucosal and cellular compartments using p-NF-κB and p53 staining. The nuclear p-NF-κB in treated-HM was

expressed as the positive nuclei to the total number of nuclei (defined as nuclear positivity), while the total p53 protein levels were expressed as the positive total number of cells (defined as total positivity) derived from two independent images per tissue section (at least four tissue sections per group) (mean ± SD using multiple *t*-tests).

1.4. Quantitative Real-Time PCR

Quantitative real-time PCR analysis (Bio-Rad real-time thermal cycler CFX96™) was performed to determine the transcriptional levels of genes related to oncogenic pathways, as we had previously characterized in our prior in vitro and in vivo descriptions [16,17,19,21,22,59,69]. Total RNA was isolated from four representative murine hypopharyngeal tissue specimens of each experimental and control group (from two males and two females) using an RNeasy mini kit (Qiagen®, Germantown, MD, USA) perform real-time qPCR. RNA quality was determined via absorption ratios at 260/280 nm (>2.0) and concentration ratios via absorption at 260 nm using a NanoDrop™ 1000 spectrophotometer (Thermo-Scientific). Reverse transcription to cDNA was performed using an iScript cDNA synthesis kit (Bio-Rad), according to the manufacturer's instructions. The real-time qPCR analysis (Bio-Rad real-time thermal cycler CFX96™) was performed using specific primers for the mouse genome, as indicated in Table S2, and iQ™ SYBR® Green Supermix (Bio-Rad). The target genes included *Rela*, *Egfr*, *Tnf*, *Bcl2*, *Il6*, and *Wnt5a*, while *Gapdh* (Glyceraldehyde 3-phosphate dehydrogenase) was used as the reference control gene (QuantiTect® primers assay, Qiagen®). The PCR assays were performed in 96-well plates and each sample was assayed in triplicate. PCR data were analyzed using CFX96™ software (Bio-Rad). Relative mRNA expression levels were estimated for each target gene relative to the reference genes ($\Delta\Delta$Ct).

1.5. miRNA Analysis

We performed miRNA analysis to determine the expression levels of "oncomir" and "tumor suppressor" miRNA specific markers in HM exposed for 45 and 100 days. Specifically, we analyzed the expression of "oncomirs" *miR-21*, *miR-155, and miR-192*, and "tumor suppressors" *miR-34a*, *miR-375*, and *miR-451a*, previously linked to laryngopharyngeal cancer [17,32–40], using primers for target miRNAs of the mouse genome (miScript Primer Assays, Qiagen®) and small normalization control RNA snRNA *RNU6B* (*RNU6-2*), as previously described [17,18,20,22] (Table S3). Briefly, a miScript II RT kit (Qiagen®) was used to perform reverse transcription synthesis of miRNAs from total RNA (isolated for qPCR analysis as described above) according to the manufacturer's instructions, and we estimated the relative expression levels (target miRNA/RNU6B) for each specific miRNA marker in each experimental and control group (CFX96™ software; Bio-Rad).

1.6. Statistical Analysis

Statistical analysis was performed using GraphPad Prism 7.0 software and one-way ANOVA (Friedman or Kruskal–Wallis; Dunn's multiple analysis test; p-values < 0.05), as well as *t*-test analysis (multiple comparisons using Holm–Sidak) to reveal any evidence of statistically significant reductions of protein, mRNA, or miRNA expression levels among the analyzed groups. We performed Spearman non-parametric correlations to estimate the correlation coefficient between expression levels of the analyzed markers in the studied groups (p-values < 0.05).

2. Supplementary Data

2.1. Micro-Invasion of Neoplastic Cells Was Identified in 45 Days of Acidic-Bile-Exposed HM

We previously demonstrated that 45 days of exposure of murine hypopharyngeal mucosa (HM) to acidic bile induced abnormal hyperplasia and moderate-to-severe dysplasia [16]. In the current experimental study, we also displayed results from 45 days of exposure in parallel to confirm our previous observations and to establish the progression of molecular and histologic changes relative to the time of exposure. We observed that 45 days of acidic bile exposure produced early micro-

invasion (Figure 1A). This phenotype was characterized by cyto-architectural changes extending into the upper layers of the mucosa and mitotic figures throughout the depth of the mucosa, accompanied by increased cell proliferation rates documented by Ki67, as well as elevated NF-κB positivity, compared to saline-treated HM (Figure 2A). Micro-invasion was evidenced by breaching the basement membrane while maintaining full-thickness nuclear hyperchromatism without surface maturation. Scoring for nuclear positivity of NF-κB and Ki67 showed statistically significant higher levels in acidic-bile-treated HM with dysplastic lesions or micro-invasion compared to normal mucosa (Figure 2B).

IHC analysis also revealed an intense staining for all DNA damage markers throughout the dysplastic epithelium in HM exposed to acidic bile for 45 days (Figure 3A), supporting the understanding that acidic bile-induced DNA damage was an early event during the neoplastic progression. IHC analysis for E-Cadherin also revealed weak staining in HM treated with acidic bile for 45 days compared to the saline-treated controls (Figure 3A).

2.2. Correlation Analysis

A Spearman nonparametric test demonstrated (i) strong positive correlations betweenthe nuclear positivity of p-NF-κB and p53 (r = 0.8286, p= 0.0292), p-NF-κB and cell proliferation markers (r = 1, p = 0.0003), and p-NF-κB and oxidative damage markers (r = 0.8286, p = 0.0292). (ii) We also found a positive correlation, although not statistically significant, between the nuclear positivity of p-NF-κB and γH2AX (r = 0.7143, p = 0.0681). (iii) Strong positive correlations were also found between cell proliferation markers Ki67 and CK14 (r = 1, p = 0.0028), (iv) cell proliferation markers and p53 (r = 0.8857, p = 0.033), (v) oxidative damage markers and γH2AX (r = 0.9428, p = 0.016), (vi) oxidative damage markers and cell proliferation markers (r = 0.9428, p = 0.016), (vii) oxidative damage markers and p53 (r = 0.9428, p = 0.016), γH2AX and cell proliferation markers (r = 1, p = 0.0027), and (viii) γH2AX and p53 (r = 0.8857, p = 0.033).

Correlation analysis also revealed inverse linear correlations, although not statistically significant, between cell–cell adhesion marker E-Cadherin and p53 (r = 0.8285, p = 0.058), E-Cadherin and oxidative damage markers (r = 0.71, p = 0.13), E-Cadherin and γH2AX (r = 0.54, p = 0.29), and between E-Cadherin and cell proliferation markers (r = 0.54, p = 0.29).

Statistical analysis also revealed a significant positive correlation between bile-induced (i) transcriptional levels of *Rela* and *Stat3* (r = 0.9429, p = 0.016), *Egfr* and *Stat3* (r = 0.82857, p = 0.029), *Egfr* and *Wnt5a* (r = 0.94285, p = 0.016), *Bcl2* and *Il6* (r = 0.8857, p = 0.033), *Stat3* and *Wnt5a* (r = 0.9418, p = 0.0166), and (ii) "tumor suppressor" miRNA levels, *miR-451a* and *miR-34a* (r = 0.943, p = 0.017).

Table S1. Percentage (%) of C57BL/6J mice exhibiting histopathological alterations of hypopharyngeal mucosa (HM) after 45 and 100 days of treatment.

C57Bl/6J mice	45-day Treated HM			100-day Treated HM		
	Saline (pH 7.0)	Acid (pH 3.0)	Bile (pH 3.0)	Saline (pH 7.0)	Acid (pH 3.0)	Bile (pH 3.0)
Total analyzed (% surv./total)	100% (4/4)	87.5% (3/4)	100% (4/4)	100% (4/4)	100% (4/4)	100% (4/4)
Hyperplasia/Dysplasia/ Micro-invasion (% obser./surv.)	0% (0/4)	0% (0/3)	100% (4/4)	0% (0/4)	0% (0/4)	100% (4/4)
Malignant lesion (% obser./surv.)	0% (0/4)	0% (0/3)	0% (0/4)	0% (0/4)	0% (0/4)	50% (2/4)

Table S2. Mouse genes (target and reference *Gapdh*) and their detected transcripts, analyzed using real-time qPCR, in murine HM.

Gene (Mouse)	Detected Transcripts	Amplicon Length (bp)
Gapdh	NM_008084	144
	NM_001289726	
Rela	NM_009045	82
Stat3	NM_011486	99
	NM_213659	
	NM_213660	
Wnt5a	NM_001256224	130
	NM_009524	
Bcl2	NM_009741	80
Tnf	NM_013693	112
	NM_001278601	
Egfr	NM_007912	68
	NM_207655	
Il6	NM_031168	128

Table S3. Mouse mature miRNAs (targets) and reference. *RNU6-2* small RNA control, analyzed using real-time qPCR, in murine HM.

miRNA (Mouse)	Target Mature miRNA, Sanger Accession)
miR-21a	mmu-miR-21a-5p, MI0000569
miR-155	mmu-miR-155-5p, MI0000177
miR-192	mmu-miR-192-5p, MI0000551
miR-375	mmu-miR-375-3p, MI0000792
miR-34a	mmu-miR-34a-5p, MI0000584
miR-451a	mmu-miR-451a, MI0001730
**Small RNA**	**Control**
RNU6	U6 small nuclear RNA, ENSMUSG00000095132

Table S4A. The 45-day and 100-day acidic-bile-induced transcriptional levels of the NF-κB-related oncogenic pathway in murine HM.

Target Gene/*Gapdh* ($\Delta\Delta^{CT}$)	45 days			100 days		
	Saline pH 7.0	Acid pH 3.0	Acidic Bile pH 3.0	Saline pH 7.0	Acid pH 3.0	Acidic Bile pH 3.0
Bcl2	1.69×10^{-2}	9.79×10^{-3}	4.35×10^{-2}	1.09×10^{-3}	6.06×10^{-4}	3.00×10^{-2}
Rela	3.70×10^{-3}	2.67×10^{-3}	6.07×10^{-3}	4.04×10^{-3}	6.84×10^{-3}	6.29×10^{-2}
Egfr	1.40×10^{-4}	8.70×10^{-5}	1.87×10^{-3}	2.38×10^{-3}	1.67×10^{-3}	9.78×10^{-2}
Tnf	1.03×10^{-3}	1.81×10^{-4}	1.69×10^{-3}	2.90×10^{-4}	2.30×10^{-4}	3.25×10^{-2}
Il6	1.20×10^{-4}	4.39×10^{-5}	3.20×10^{-4}	1.41×10^{-5}	2.04×10^{-5}	1.47×10^{-3}
Stat3	2.00×10^{-4}	1.22×10^{-4}	2.95×10^{-3}	4.27×10^{-3}	4.90×10^{-3}	9.01×10^{-2}
Wnt5a	3.40×10^{-4}	3.05×10^{-4}	4.58×10^{-4}	7.48×10^{-4}	4.96×10^{-4}	9.80×10^{-4}

Table S4B. Relative mRNA expression ratios for each target gene in murine HM exposed to acidic bile for 45 and 100 days relative to the corresponding saline-treated controls.

Target Gene/*Gapdh* ($\Delta\Delta^{CT}$)	45 days of Acidic Bile/Control*	100 days of Acidic Bile/Control
Bcl2	2.6	27.6
Rela	1.6	14.3
Egfr	13.4	41.0
Tnf	1.6	112.2
Il6	2.7	104.6
Stat3	14.8	21.1
Wnt5a	1.3	1.3

Table S5A. The 45-day and 100-day acidic-bile-induced miRNA levels in exposed murine HM.

Target miRNA/*RNU6** ($\Delta\Delta^{CT}$)	45 days			100 days		
	Saline pH 7.0	Acid pH 3.0	Acidic Bile pH 3.0	Saline pH 7.0	Acid pH 3.0	Acidic Bile pH 3.0
miR-21	2.77×10^{-2}	1.43×10^{-3}	8.23×10^{-2}	8.88×10^{-3}	5.12×10^{-2}	6.69×10^{-2}
miR-155	3.27×10^{-3}	3.17×10^{-3}	7.86×10^{-3}	2.20×10^{-3}	1.63×10^{-2}	6.40×10^{-2}
miR-192	1.32×10^{-2}	3.25×10^{-3}	1.83×10^{-2}	2.04×10^{-2}	1.00×10^{-2}	6.67×10^{-2}
miR-375	6.80×10^{1}	5.79×10^{1}	4.60×10^{1}	7.60×10^{1}	6.29×10^{1}	0.44×10^{1}
miR-34a	5.68×10^{-1}	7.01×10^{-2}	6.05×10^{-2}	3.60×10^{-2}	2.67×10^{-2}	2.33×10^{-2}
miR-451a	7.60×10^{-3}	5.21×10^{-3}	2.29×10^{-3}	4.66×10^{-3}	8.00×10^{-4}	6.46×10^{-4}

* Normalization of miRNA levels using small RNA (snRNA RNU6B (RNU6-2)).

Table S5B. Relative miRNA expression ratios for each marker in murine HM exposed to acidic bile for 45 and 100 days relative to the corresponding saline-treated controls.

Target miRNA/*RNU6* ($\Delta\Delta^{CT}$)	45 days of Acidic Bile/Control*	100 days of Acidic Bile/Control
miR-21	3.0	7.5
miR-155	2.4	29.1
miR-192	1.3	3.3
miR-375	0.7	0.1
miR-34a	0.1	0.6
miR-451a	0.3	0.1

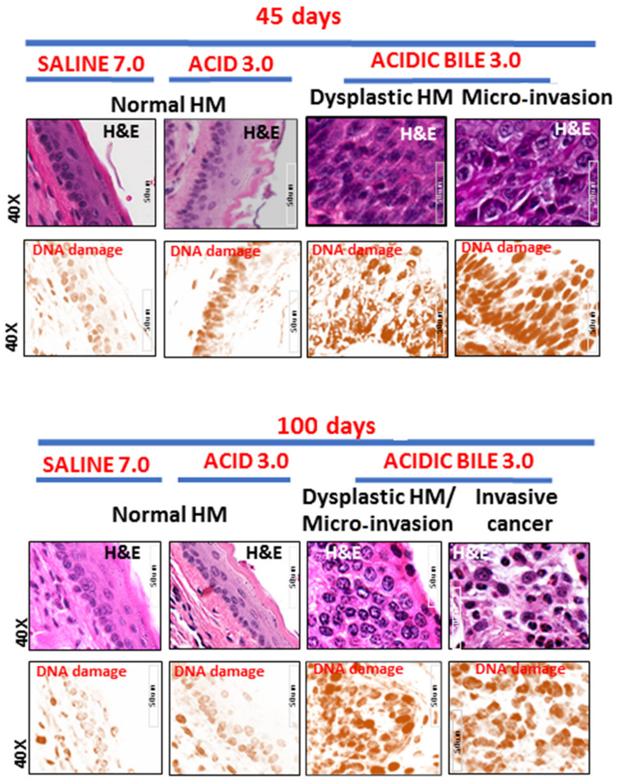

Figure S1. Acidic bile-induced chromogenic immunohistochemical staining for DNA damage at 45 days and 100 days of exposure**.** Immunohistochemical analysis for γH2AX and DNA/RNA oxidative damage markers was performed using chromogenic staining (brown) (H&E: hematoxylin and eosin staining).

© 2020 by the authors. Licensee MDPI, Basel, Switzerland. This article is an open access article distributed under the terms and conditions of the Creative Commons Attribution (CC BY) license (http://creativecommons.org/licenses/by/4.0/).
